# Towards developing guidelines and systems of care to facilitate early reperfusion for ST -elevation myocardial infarction in Africa

**Published:** 2014

**Authors:** Rhena Delport

**Affiliations:** Department of Chemical Pathology, University of Pretoria, Pretoria, South Africa

## Outline

The theme for the 15th annual SA Heart Congress for 2014, ‘Bridging the divide’ between best practice and current challenges in the management of cardiovascular conditions, inspired this editorial on the feasibility of implementing the European Society of Cardiology ‘Stent-for-Life’ initiative in sub-Saharan Africa or alternate measures of ensuring early reperfusion for myocardial ischaemia. This editorial explores the changing burden of non-communicable diseases (NCD) in Africa that impact on the occurrence of ST-elevation myocardial infarction (STEMI) in Africa, revisits international guidelines on early reperfusion and implementation of systems of care, and identifies factors related to timely myocardial reperfusion in remote areas.

## Current status in Africa

Recent comments by Kengne and Mayosi on the rising incidence of chronic NCD in sub-Saharan Africa in both rural and urban areas express concern about the lack of preparedness of African states for the pending pressure on healthcare services,^1^ pertain to South African healthcare services as well.^2^,^3^ The World Health Organisation (WHO) estimates that NCDs will exceed communicable diseases as the leading cause of death in Africa in 2030.^4^,^5^

An increase in cardiovascular disease (CVD) burden is also expected due to the increased prevalence and incidence of CVD risk factors, paucity of surveillance data and registries, lack of interventional measures, as well as a shortage of physicians and cardiologists, inadequate diagnostic capabilities, and misguided opinions.^6^-^8^ Although CVD remains the leading cause of death in the world,^9^ three-quarters of which occur in low- and middle-income populations,6 the burden of ischaemic heart disease (IHD) remains low in comparison with other causes of heart disease, particularly in people of African descent.^6^,^10^,^11^ Marked variability is however observed in the incidence, prevalence and mortality rates of IHD across developing countries, as in Africa, mainly due to the differences in composition and severity of risk factors and management thereof, as well as the stage of epidemiological transition.^6^,^11^-^20^

Concerted action among the WHO and international cardiac societies to improve cardiovascular health and prevent death from cardiovascular disease is increasingly becoming evident.^21^,^22^ Hopefully African societies will follow suit.

## Current guidelines

The majority of recommendations in the European^23^ and American^24^ guidelines for the management of STEMI were perceived as either identical or overlapping.^25^ The detail of the guidelines will not be replicated here, neither is the aim of this editorial to perform further comparisons with other international guidelines. A brief exposition on primary reperfusion strategies will be provided from random sources, with the emphasis on the African context where percutaneous coronary intervention (PCI) facilities are sparsely distributed and emergency medical services (EMS) are not readily available.

Primary percutaneous coronary intervention is the preferred and most effective option for reperfusion, provided that the intervention is performed timely by an experienced operator.^26^,^27^ Although performance metrics such as ‘door-to-balloon time’ or ‘door-to-needle time’ are employed to quantify time lapses from the onset of symptoms to definitive treatment, the concept of ‘first medical contact (FMC)-to-device time’ recognises the need for speedy diagnosis and treatment as the primary outcome.^26^

The patient as well as factors relating to EMS determine the time delay between the onset of symptoms and the FMC, while FMC and the beginning of reperfusion is explained by EMS transport time to a PCI-capable facility and determinants of ‘door-to-balloon’ time.^27^ Ideally the patient should be transported directly to a PCI-capable hospital for primary PCI but if the patient is admitted to a non-PCI facility, the door-in-door-out time should ideally be 30 minutes or less before the patient is transported to a PCI-capable hospital.^26^ The FMC-to-device time should be 90 minutes or less, and in the case of necessity to transfer the patient for PCI, 120 minutes or less. If primary PCI is not achievable within 120 minutes thrombolytics should be administered with FMC within 30 minutes of diagnosis of STEMI either pre-hospital by a trained paramedic/clinic nurse, or, alternatively, by a physician in the nearest ER.^26^,^27^

Additional recommendations of relevance as proposed in the United Kingdom ‘NICE’ guidelines^28^ entail the following (as quoted):

• Offer coronary angiography, with follow-on PPCI if indicated, as the preferred coronary reperfusion strategy for people with acute STEMI if:
– Presentation is within 12 h of onset of symptoms and
– PPCI can be delivered within 120 min of the time when fibrinolysis could have been given.
• Offer fibrinolysis to people with acute STEMI presenting within 12 h of onset of symptoms if PPCI cannot be delivered within 120 min of the time when fibrinolysis could have been given.
• Consider coronary angiography, with follow-on PPCI if indicated, for people with acute STEMI presenting more than 12 h after the onset of symptoms if there is evidence of continuing myocardial ischaemia.
• Offer coronary angiography, with follow-on PPCI if indicated, to people with acute STEMI and cardiogenic shock who present within 12 h of the onset of symptoms of STEMI.
• Offer an ECG to people treated with fibrinolysis, 60–90 min after administration. For those who have residual ST-segment elevation suggesting failed coronary reperfusion:
– Offer immediate coronary angiography, with follow-on PCI if indicated
– Do not repeat fibrinolytic therapy.
• If a person has recurrent myocardial ischaemia after fibrinolysis, seek immediate specialist cardiological advice and, if appropriate, offer coronary angiography, with follow-on PCI if indicated.
• When commissioning PPCI services for people with acute STEMI, be aware that outcomes are strongly related to how quickly PPCI is delivered, and that they can be influenced by the number of procedures carried out by the PPCI centre.

Factors that may contribute to earlier treatment for PCI-treated patients include bypassing non-PCI-capable hospitals and bypassing the emergency department of the PCI-capable hospital, pre-hospital ECG diagnosis of STEMI, and pre-hospital activation of the catheterisation laboratory by emergency physician or EMS, and early (within 20 minutes) activation of the catheterisation laboratory team.^27^,^28^

## Current guidelines for remote areas

For the treatment of STEMI patients living in remote, sparsely populated areas with no ready access to PCI facilities, the pharmaco-invasive strategy is advocated. Fibrinolysis should be commenced as soon as possible if there are no contraindications, followed by transfer to a PCI facility for rescue PCI or angiography with possible PCI as a routine measure. Patients with contra-indications for fibrinolysis, late presenters, and patients with cardiogenic shock should be transferred to a PCI facility irrespective of the duration of transfer. Clear treatment protocols and a well-organised STEMI network are pivotal in STEMI management in these areas.^29^

From the Australian experience, we learn that direct transport to PCI facilities and inter-hospital transfer for primary PCI positively impact on timely access to primary PCI (defined as ‘the proportion of the population capable of reaching a PPCI facility ≤ 120 minutes from emergency medical services activation’) and that pre-hospital fibrinolysis significantly improves timely access to reperfusion PCI (defined as ‘the proportion of the population capable of reaching a fibrinolysis facility in ≤ 60 minutes from emergency medical services activation’) in remote areas.^29^ Geographical information systems were employed to integrate hospital, classified as hospitals that provided PCI or fibrinolysis, and population and road network data,^30^ which in all probability contributes to informed management of STEMI care.

## Concluding remark

In our endeavour to facilitate early reperfusion for ST elevation myocardial infarction in Africa we need to bear in mind that *‘Improvements in access to timely care for patients with STEMI will require a multifaceted approach involving patient education, improvements in the emergency medical services and emergency department components of care, the establishment of networks of STEMI-referral hospitals (not PCI capable) and STEMI-receiving hospitals (PCI capable), as well as coordinated advocacy efforts to work with payers and policy makers to implement a much-needed healthcare system redesign. By focusing now on system efforts for improvements in timely care for STEMI, we will complete the cycle of research initiated by Reimer and Jennings 30 years ago. Time is muscle . . . we must translate that into practice’* (Elliott M Antman, 2008).^31^
